# A marine sponge associated fungal metabolite monacolin X suppresses angiogenesis by down regulating VEGFR2 signaling†

**DOI:** 10.1039/c9ra05262c

**Published:** 2019-08-27

**Authors:** Sirpu Natesh Nagabhishek, Arumugam Madan Kumar, Sambhavi B., Anandan Balakrishnan, Yash T. Katakia, Suvro Chatterjee, Nagarajan Nagasundaram

**Affiliations:** Cancer Biology Lab, Molecular and Nanomedicine Research Unit, Sathyabama Institute of Science and Technology Chennai-600 119 Tamil Nadu India madankumarbio@gmail.com madankumar@sathyabama.ac.in +91 9942110146; Department of Genetics, Dr ALM PGIBMS University of Madras Taramani Chennai Tamil Nadu India; Vascular Biology Lab, AU-KBC Research Centre, Department of Biotechnology, Anna University Chennai Tamil Nadu India; School of Humanities, Nanyang Technological University 14 Nanyang Dr Singapore

## Abstract

Cancer is one of the leading causes of global death and there is an urgent need for the development of cancer treatment; targeting VEGFR2 could be one of the promising therapies. In the present study, previously isolated marine fungal metabolite monacolin X, suppresses *in vitro* angiogenic characteristics such as proliferation, migration, adhesion, invasion and tube formation of HUVECs when stimulated by VEGF, at a non-toxic concentration. Monacolin X downregulated VEGFR2, PKCα and PKCη mRNA expression. Further, monacolin X inhibited *in vivo* angiogenesis in CAM assay, vascular sprouting in aortic ring, decreased ISV and SIV length and diameter in Tg (Kdr:EGFP)/ko1 zebrafish embryos. Monacolin X showed reduced protein expression of pVEGFR2, pAKT1, pMAPKAPK2, pFAK and pERK1 in breast cancer lines and in DMBA induced mammary carcinoma in SD rats showed tumor regression and anti-angiogenesis ability *via* decrease pVEGFR2 and pAKT1 protein expression. *In silico* studies also revealed monacolin X ability to bind to crucial amino acid Cys 919 in the active site of VEGFR2 suggesting it to be a potent VEGFR2 inhibitor.

## Abstract 2

Cancer is one of the leading causes of global deaths and there is an urgent need for the development cancer treatment; targeting VEGFR2 could be one of the promising therapies.

## Graphical abstract

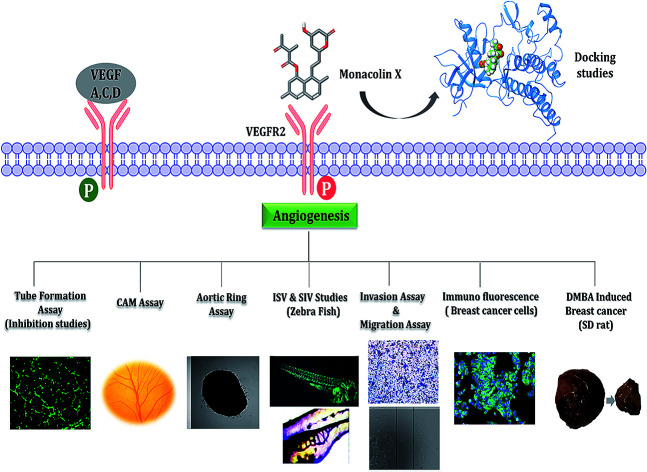

## 1 Introduction

Angiogenesis is crucial/essential for normal vasculature, defined as development of new capillaries from the existing blood vessels which plays a pivotal role during ontogenic development and is also essential for various physiological processes like tissue repair and growth.^1^ Angiogenesis is a very complex process consisting of migration, proliferation and differentiation of endothelial cells regulated with several growth factors, specific receptors and many intracellular signaling pathways.^2^ However uncontrolled vessel growth contributes to malignant tumor growth, metastasis, eye disease, inflammatory disorders and ischemic heart diseases. Angiogenesis inhibitors will avert the formation of a new blood vessel which in turn stops or decelerates the growth or spread of a tumor.^3^ Thus, blocking blood supply to a specific region is an attractive therapeutic strategy for the treatment of a wide variety of human diseases.^4,5^ Angiogenesis process can be inhibited in different ways like-by blocking the angiogenesis growth factors like VEGF (Vascular Endothelial Growth Factor), by blocking the cell tyrosine kinase activation or a VEGFR inhibitors, by acting on the chemical messengers that cells use to signal to each other to grow,^6^ by inhibiting endothelial cells growth, and preventing extracellular matrix breakdown.^7^ Primary tumor growth and metastasis are two such events which involve a crucial event of tumor angiogenesis and hence, anti-angiogenic treatment of tumors is a highly promising therapeutic approach.^8^

The VEGF is a major noticeable growth factor which plays a predominant role in angiogenesis and has high affinity towards its tyrosine kinase receptors (VEGFR-1, VEGFR2 & VEGFR-3).^9^ VEGFR1 and VEGFR2 mainly help in vasculogenesis, where VEGFR3 regulated in lymphogenesis. VEGFR2 kinase activity is ten folds stronger than VEGFR1 and helps in a great extent throughout angiogenesis, a prime receptor in transmitting angiogenic signals.^10^ Therefore, inhibiting VEGFR2 decrease angiogenesis and block VEGFR2 signaling cascade providing potential approach to develop anti-angiogenesis therapies.^11^

Marine species have been less explored for bioactive molecules for pharmaceutical use and are a greater source for identifying newer drugs which can target various diseases.^12^ Our earlier study investigated with monacolin X a secondary metabolite isolated from fungi-NMK7 associated with marine sponge showed antiproliferative/cytotoxic, antimigratory, apoptotic inducing ability on different breast cancer and normal cell lines.^13^ However, there is no clear evidence on antiangiogenic activity. Therefore, in this study, we have evaluated the antiangiogenic activity of monacolin X using *in vitro*, *in vivo* and *in silico* methods.

## 2 Materials and methods

### 2.1. Reagents and chemicals

All chemicals were purchased from Sigma-Aldrich (USA) and Himedia Laboratories Pvt. Ltd India, unless stated otherwise. Cell culture plastics were from Tarsons Products (P) Ltd India.

### 2.2. Cell line and culture

HUVECs cell culture was purchased from Himedia and maintained at molecular biology lab Madras University India. The cells were grown in T25 culture flasks containing HiEndoXL™ Endothelial Cell Expansion Medium (AL517) using 2% serum and supplemented with endothelial cell expansion supplement growth factors provided in the kit and 1% antibiotics (Gentamicin and Amphotericin B). Cells were maintained at 37 °C in a humidified atmosphere containing 5% CO_2_. Upon reaching confluence, the cells were trypsinized using EnVzyme. Endothelial cells EA.hy926 were obtained from vascular biology lab MIT campus Anna University. Human umbilical vein cell lines were established by fusing primary human umbilical vein cells with a thioguanine-resistant clone of A549 by polyethylene glycol (PEG) exposure. Iscove's Modified Dulbecco's Medium (IMDM, Biological Industries, Israel) supplemented with 10% v/v FBS, 100 U mL^−1^ penicillin, and 100 mg mL^−1^ streptomycin at 37 °C and 5% CO_2_ was used for cell maintenance and growth.

### 2.3. Cell viability of monacolin X on EA.hy926 and HUVECs by WST method

Antiproliferative effect of monacolin X at various concentrations was studied on human endothelial cells EA.hy926 and HUVECs cell culture. The cells were grown in T25 culture flasks as mentioned above and upon reaching confluence, the cells were detached using Trypsin–EDTA solution and were sub-cultured at a density of 5000 cells per well. At 50% confluence, the culture medium was aspirated and cells were treated with different concentrations (0, 10, 20, 40, 80, 150, 250 and 300 μM) of monacolin X for 24 h at 37 °C in the CO_2_ incubator. Later cells were incubated with WST-8 (2-(2-methoxy-4-nitrophenyl)-3-(4-nitrophenyl)-5-(2,4-disulfophenyl)-2*H*-tetrazolium), as per Cell Counting Kit-8 method by Sigma was measured at 450 nm with a standard microplate reader (Enspireperkin Elimer USA).
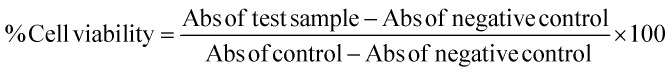


### 2.4. Cellular integrity measurement by LDH assay

Cell membrane integrity of endothelial cells EA.hy926 and HUVECs cell culture were evaluated by determining the activity of lactate dehydrogenase (LDH) leaking out of the cell according to the manufacturer's instructions (Pierce Thermo scientific USA). The cytotoxicity was assessed quantitatively by measuring the activity of LDH in the supernatant. Briefly, cells were exposed to different concentrations of monacolin X for 24 h, and the assay was further proceeded based on our previous works^13^ then 100 μL per well of each cell-free supernatant was transferred in triplicates into wells in a 96-well plate, and 100 μL of LDH assay reaction mixture was added to each well. After 3 h incubation under standard conditions, the optical density of colour generated was determined at a wavelength of 490 nm using Multimode plate reader (EnSpire PerkinElmer USA).

### 2.5. Apoptotic studies

Cell and nuclear morphology have been evaluated using PI and AO/EB staining. The nuclear morphology was analyzed using bright field microscopy in HUVECs cells after treating with monacolin X at its IC_50_ concentration and SU5416 (10 μM) for 24 h respectively. Control cell was grown in the same manner without monacolin X. The cell were trypsinized and fixed with ethanol, then, the cell nuclei were stained using 1 mg mL^−1^ propidium iodide (PI) at 37 °C for 15 min in the dark. Further characteristic apoptotic changes were determined by AO/EB staining.^13^ Coverslips were taken and kept on glass slides and stained with 100 μL of dye mixture (1 : 1 of AO and EB) and immediately viewed under an inverted fluorescence microscope (EVOS FL digital inverted fluorescence microscope (AMG)).

### 2.6. Tube formation assay

Abcam *in vitro* Angiogenesis Assay Kit (ab204726) was used to asses the tube inhibition capacity of monacolin X. Prior to the assay, the endothelial cell tube formation matrigel was thawed at 4 °C overnight and each well of 15 ibidi μ-Slide was pre-chilled and coated with 10 μL of matrigel and incubated for 15 min at 37 °C. 1 × 10^4^ of HUVECs were added to ECMG coated well and cell was allowed to settle down and monacolin X were added at with various concentrations (15 μM, 30 μM and 60 μM) with media containing 20 ng mL^−1^ of VEGF and SU5416 (10 μM) as a standard antiangiogenic drug. After 4–12 h of incubation at 37 °C, 5% CO_2_, endothelial cell tube formation was assessed with an EVOS-FL inverted microscope with attached digital camera. Tubular structures were quantified in low power fields (4×), and the inhibition percentage was expressed using untreated wells as 100%. For quantification total tube length, total branching points, total loops and covered areas were quantified using Wim Tube software.^14^

### 2.7. Gene expression through-monacolin X treatment on HUVECs

Monacolin X was administered to HUVECs at IC_50_ concentration and SU5416 (4 μM) for a duration of 12 h in the presence of TPA at a concentration of 10 nmol L^−1^ for 4 h. The total RNA was isolated from HUVECs after 6 h of incubation with monacolin X. The amplification was performed using Clonetech SYBR Premix using the following set of primers: VEGFR2 forward: 5′-AGGAAGTAGCCGCATTTG-3′, reverse: 5′GGAGAAGACACAGACACA-3′; PKCα forward: 5′-TGGCAAAGGAGCAGAGAACT-3′, reverse: 5′-TGTAAGATGGGGTGCACAAA-3′; PKCη forward: 5′-AGTAGA CTGGTGGGCAATGG-3′, reverse: 5′-GATCCCTGTGG CATCTTCAT-3′; β-actin: forward: 5′-CTCTTCCAGCCTTCCTTCCT-3′, reverse: 5′-AGCACTGTGTTGGCGTACAG-3′. The PCR amplification was performed using qPCR (Applied Biosystem) under the following conditions: 40 cycles at 95 °C for 1 min, 61 °C for 1 min, and 74 °C for 2 min followed by 10 min at 74 °C. qPCR data were quantitatively analyzed by using the formation of 2-ΔΔCt. The relative expression levels of the mRNAs of the target genes were normalized using the β-actin internal standard.^15^

### 2.8. Aortic ring assay

Chick embryos were removed from the eggs on the 11^th^ day under sterile conditions to remove aorta and the below mentioned steps were followed-the aortic arches were cut from the heart using sterile scissors and forceps, and washed several times in 1× PBS (phosphate buffered saline). A thin layer of matrigel was coated on the coverslips placed in 24 well flat bottom cell culture plates. Aortic arches were cut into small rings of similar size (approx. 1 mm thick) with a sterile surgical blade. Following 1× PBS wash, the rings were embedded into the matrigel layer. After 3 h, IC_50_ concentration of monacolin X were added with DMEM (Dulbecco's modified Eagle's medium) and SU5416 (4 μM) to the explants and incubated at 37 °C (5% CO_2_). Further, the explants were regularly observed under the microscope to record the number of sprouts and length of capillary sprouts (μm) were analysed using Wim Sprout software.^16–18^

### 2.9. Chicken chorioallantoic membrane (CAM) assay

The effect of monacolin X on angiogenesis was evaluated using the CAM assay, following a method described previously with minor modifications.^16^ Fertilized chicken eggs were kept in a humidified egg incubator at 37 °C. The eggs were positioned horizontally and rotated several times. After 4 days of incubation, a 1 cm^2^ window was carefully created on the broad side of the egg to assess the extent of embryonic blood vessels. The normal development was verified, and embryos with malformations or dead embryos were excluded. Then, about 2 mL of albumen was aspirated from each egg through the small window. After removal of albumen, monacolin X (15 μM, 30 μM and 60 μM) on a small disc filter paper was directly placed on the small window created before. At least five eggs were used for each dose. The control group was treated with albumen (100 μL per egg), while positive control group was treated with 20 ng mL^−1^ of VEGF. After treatment, each egg was observed under a Zeiss Stemi 2000-c microscope equipped with Axiocam MRc 5 Zeiss, and blood vessels were photographed. The antiangiogenic effects of monacolin X on the CAMs were quantified Mean vessel area as a percentage of the total area, mean vessel length and mean the number of branch points which were marked using Wimcam software.^19^

### 2.10. Zebrafish husbandry

The transgenic zebrafish embryos were provided by Dr Hitoshi Okamoto (RIKEN Center for Brain Science Japan), which were acquired by spawning of Tg(Kdr:EGFP)/ko1 transgenic line naturally, which expresses green fluorescent protein (GFP) under vascular endothelial growth factor receptor 2 (vegfr2/kdr/flk1) promoter. Multiphase filtration stand-alone zebrafish systems (Danio reio Zebrafish maintenance system by aquaneering) was used for the maintenance with temperature control of 28.5 °C, on a restricted photoperiod of 14/10 h (light/dark). The process of husbandry of the zebrafish embryos and their treatment was efficiently described earlier.^20^

### 2.11. Assessment of ISV using Tg(Kdr:EGFP)/ko1 zebrafish embryos

Monacolin X was exposed to zebrafish embryos at 6–8 h post fertilization (hpf) period for 48 h at a concentration of 0.3 nM, 3 nM, 6 nM, 0.375 μM, 0.5 μM, 0.75 μM, 1 μM, 2 μM, and 10 μM and SU5416 (4 μM) in embryo medium (0.39 Danieau's solution containing 0.23 mM KCl, 19.3 mM NaCl, 0.13 mM MgSO_4_, 1.7 mM HEPES, 0.2 mM Ca (NO_3_)_2_, pH 7.0). Triplicates of 10 embryos were placed per well (*n* = 30). The embryos at 16 h exposure were observed for morphological and blood vessel development changes. Tg(Kdr:EGFP)/ko1 transgenic zebrafish embryos with fluorescent blood vessels was used to facilitate image analysis. The embryo medium with 0.003% 1-phenyl-2-thiourea (PTU) medium inhibited the pigment formation and was later incubated for 24 h post which the changes in the blood vessel development was imaged at 48 hpf using a leica M165 FC Fluorescence stereo Microscope. A monitored exposure of the embryos for 40 h was carried out in another experiment where the embryos were observed for abnormalities morphologically and intersegmental vessel abnormalities (ISV). Using an NIH ImageJ software (NIH), the length and diameter of the ISV in the embryos were measured which in turn depicted the inhibition of angiogenesis on monacolin X treatment.^15,21^

### 2.12. Assessment of SIV by ALP staining method using zebrafish embryos

Monacolin X was exposed to zebrafish embryos at 6–8 h post fertilization (hpf) period for 48 h at a concentration 0.5 μM. Alkaline phosphatase staining in zebrafish embryos was performed based on previous works done by G.N. Serbedzija *et al.* 2000 ^22^ 3dpf, embryos were dechorionated with protease and fixed in 4% paraformaldehyde for 2 h at room temperature and stained for endogenous alkaline phosphatase activity. Embryos were washed two times in phosphate buffered saline (PBS) and dehydrated by immersing in 25, 50, 75 and 100% methanol in PBT to permeabilize. Embryos were then rehydrated stepwise to 100% PBT. For staining, embryos were equilibrated in NTMT buffer (0.1 M Tris ± HCl pH 9.5; 50 mM MgCl; 0.1 M NaCl; 0.1% tween 20) at room temperature. Once the embryos equilibrated in NTMT, 4.5 μL of 75 mg mL^−1^ NBT and 3.5 μL of 50 mg mL^−1^ BCIP per mL were added. After staining for 10 min, all the blood vessels in the fish embryo were labeled. Addition of PBST to the embryos terminated the staining reaction post which the embryos were subjected to 5% formamide and 10% hydrogen peroxide along with PBS for a period of 20 min. This removed the endogenous melanin in the pigmented cells which facilitated complete visualizing of the vessels that were stained. Embryos were then examined on a stereo-microscope (Lecia, Germany) and imaged. Further, the number of normal ISV with full-length formation and quantification of SIV area was evaluated using the NIH Image J software.^23^

### 2.13. Transwell migration and invasion assay

Transwell migration assays were carried out using 24-well transwell chambers with 8 μm size PET membranes (Ibidi traswells 3464), HUVEC and EA.hy926 cells were starved for 24 hours, and 1 × 10^5^ cells were seeded on the top chamber. The bottom chambers were filled with endothelial media and monacolin X treated at its IC_50_ concentration and SU5416 (4 μM) as standard drug. For transwell migration assay, cells were placed in the upper chamber for 24 h and the number of migrated cells which are in bottom of the membrane is stained with crystal violet and above cells were swapped off, cells were counted in five random fields per chamber by phase contrast microscope and statistically analyzed and for transwell invasion assay the experimental procedure is the same whereas here the cells were seeded on the transwell chambers precoated with ECM Matrix gel solution.^24^

### 2.14. Wound healing assay

Wound healing assay was analyzed by using Ibidi 24 well plate with culture inserts in it. Then 70 μL of cells were added in the insert and grown to confluence, the inserts were removed to create the uniform wound of 500 μm. HUVEC were treated with monacolin X at IC_50_ concentration and SU5416 (4 μM). Post wounding, the process of the healing was documented at different time intervals through microphotographs and the numerical significance of the migrating cells was achieved by counting all the cells present within 0.4 mm region at the sight of the wound. A minimum of 3 individual cultures was used to calculate the mean wound healing capacity of each cell culture condition.

### 2.15. Immunofluorescence analysis

To check the protein expression levels of phospho forms of VEGFR2, AKT1, FAK, MAPKAPK2 and ERK1 were analysed by Immunofluorescence staining as described previously,^25^ briefly MDA-MB-231 high metastatic cells and T47D low metastatic cells were treated with IC_50_ concentration of monacolin X^13^ and SU5416 (4 μM) as a standard drug control for 24 h respectively. Later the cells were fixed in 4% paraformaldehyde for 15–30 min, at room temperature, then washed with PBS and blocked for 1 h using blocking buffer at room temperature. Cover slides were incubated with Anti-VEGFR2 (Phospho Y-1175) (Abcam-ab194806) at a dilution of 1 : 200 (rabbit polyclonal); Anti-AKT1 (Phospho S-473) (Abcam-ab81283) at a dilution of 1 : 200 (rabbit monoclonal); Anti-FAK (Phospho Y-397) (Abcam-ab81298) at a dilution of 1 : 200 (rabbit polyclonal); Anti-MAPKAPK2 (Phospho T-334) (Abcam-ab63378) at a dilution of 1 : 100 (rabbit polyclonal) and Anti-ERK1(Phospho Y-204) (Abcam-ab131438) at a dilution of 1 : 100 (rabbit polyclonal) overnight at 4 °C and washed with PBS. Appropriate fluorophore-labelled secondary antibody Goat Anti-Rabbit IgG H&L (FITC) (ab6717) was added at a dilution of 1 : 250 and incubated for 2 h at room temperature later washed with PBS, DAPI dye was added to reveal nuclear DNA. Immunofluorescence was visualized under an inverted fluorescence microscope (EVOS FL digital inverted fluorescence microscope).

### 2.16. Animal study

All the experiments, habitat and victuals were designed and strictly performed in accordance with CPCSEA guidelines (Committee for the Purpose of Control and Supervision of Experiments on Animals, Ministry of Social Justice and Empowerment, Government of India), and was approved by the Institutional Animal Ethical Committee (IAEC) (Approval No; SU/CLATR/IAEC/IV/025/2016) of Sathyabama Institute of Science and Technology Chennai India. Female SD rats (50–55 days old) were used in this study and were purchased from Biogen Bangalore and were maintained in central animal house facility, Sathyabama Institution of science and technology. Pre-experimental measures were taken place like acclimatizing the animals to the laboratory prerequisite conditions, standard parameters like temperature of 23 ± 2 °C, a 60–70% of humidity, a standard ratio of artificial light and dark (12 h:12 h) (lights on from 6 a. m., light intensity 150 lux per cage) *etc.* Throughout the experiment, the animals were given tap water as a source of drinking water and the feed was restricted to commercially available pelleted diet (M/S Hindustan Foods Ltd, Bangalore, India).

#### 2.16.1 Experimental design

The animals were divided into four groups of six animals each.

Group 1: Animals received 0.5 mL corn oil thrice a week orally for 12 weeks.

Group 2: 8 week old female SD rats were induced for mammary carcinoma by the administration of 25 mg kg^−1^ DMBA dissolved in 0.5 mL of corn oil, by oral gastric intubation of a single dose. The rats were allowed for 12 weeks for the development of a mass amount of mammary carcinoma from the day of induction.

Group 3: mammary carcinoma was induced as in group 2 and post treatment of monacolin X at 150 mg kg^−1^ d^−1^ dissolved in 0.5 mL corn oil and given at 8 cycles for 4 weeks at the site close to the tumor area. Treatment was started as soon as the tumor size reached 50 mm^3^. This group was used to study the chemotherapeutic potential of monacolin X in the experimental animals.

Group 4: animals were treated with SU5416 and was delivered subcutaneously in a centyl-methyl-cellulose (CMC) suspension 100 mg kg^−1^ every second day from the day tumors reached a volume threshold of 50 mm^3^.

After the experimental period, the animals were anesthetized using ether and sacrificed by cervical decapitation. The tissue was excised out, weighed and the part of tissue used for histopathology.^26^

### 2.17. Tumor induction and measurement of tumor parameters

For a sufficient amount of 100% tumor incidence, the SD rats were subjected to 25 mg kg^−1^ of body weight of DMBA which in turn induced mammary tumors in 8–12 weeks. After achieving a tumor of up to 50 mm^3^, animals were sacrificed. The tumor volume was calculated using, *V* = (*W*(2) × *L*)/2, where V is tumor volume, *W* is tumor width, *L* is tumor length for caliper measurements.^27^ Tumor incidence is the percentage of tumors present in a group.^28^

### 2.18. Histopathology

The breast samples were fixed in 10% formalin and embedded in paraffin wax and were evaluated for their histological nature. For the purpose of Immunohistochemistry, sections of breast sample were cut into 4 μm thickness and pathological changes of the animal tissues were observed through a light microscope in the experimental rats.

### 2.19. Immunohistochemistry (IHC)

Formalin fixed paraffin embedded tumor tissue samples were collected on silane-coated slides, and the protein expression of pVEGFR2 and pAKT1 was assessed by IHC. Immunohistochemical staining was carried out based on our previous method.^26^ The tissue sections were deparaffinized in xylene and dehydrated using graded ethanol solutions. The antigen retrieval was done by Tris–EDTA buffer. 0.3% H_2_O_2_ in methanol was subjected to observe the endogenous peroxide activity for a time period of 30 min. PBS was used as a rinsing agent and 3% BSA as a blocking solution and the activity was undertaken at room temperature for a time period of 1 h to avoid non-specific binding. The sections were then incubated with Anti-VEGFR2 (Phospho Y-1175) rabbit polyclonal (Abcam-ab194806) at dilutions of 1 : 50 and Anti-AKT1 (Phospho S-473) rabbit monoclonal (Abcam-ab81283) at dilutions of 1 : 100 at 4 °C overnight. The slides were washed with PBS and then incubated with HRP-conjugated secondary antibody, Goat Anti-Rabbit IgG H&L (HRP) (Abcam-ab97051) for 1 h at room temperature. The peroxidase activity was visualized by treating with DAB and then counterstained with Meyer's hematoxylin. Quantitative analysis was performed in a blinded manner under a light microscope. In each slide >1000 cells were counted and the percentage of cells with strong nuclear staining was depicted. All immunostained slides were analyzed by a pathologist to assess pVEGFR2 and pAkt expression in tumor vasculature and cells different scoring approaches were examined for vessels and tumor cells. An assessment of the slides were done based on the previous report by Holzer TR *et al.*, where level of intensity of tumor cell staining (range of 0, no staining; 1+, weak staining; 2+, moderate staining; 3+, intense staining) was made objectively by the study pathologist after screening entire area of the stained tissue section. The cytoplasmic and the nuclear compartments of the tumor cells were observed thoroughly for percentage of tumor cells that were being stained increased with each level of increased staining intensity making them directly proportional. The value of each staining level (0, 1, 2 or 3) was multiplied by the respective percentage of tumor cells at that intensity level and the histogram represents the number of positive cells.^29^

### 2.20. Molecular docking analysis and molecular dynamic simulation

Schrodinger suite was used to perform the *in silico* studies. The VEGFR2 crystal structure was retrieved from RCSB PDB database with the corresponding PDB ID'S 3U6J.^30^ Through protein preparation wizard the protein was refined by setting all the parameters to default.^31^ Similarly, ligands monacolin X and SU5416, chemical structure files were obtained from pubchem database (pubchem id: 125978 and 5329098) and prepared by using LigPrep module. Properties like human oral absorption, central nervous system activity, predicted brain/blood partition coefficient (QP log BB) and Lipinski rule of five of the compounds were calculated using the QikProp module.^32^ Using the receptor grid generation method the binding pocket in the receptor was fixed and into that using Glide XP docking application^33^ the molecules were docked. The complexes were analyzed using the Desmond simulations,^34^ Molecular dynamic simulations were carried using two steps one was the system builder and the next for molecular dynamic simulations. The parameters used under the system-builder step are simple point charge (SPC) water model and orthorhombic periodic boundary in the solvation tab, neutralization with 1cl^−^ counter ions and a salt concentration of 0.15 M were opted in ions tab under the OPLS 3e force field. In the molecular dynamics step, NPT as ensemble class, temperature at 300 K, 1 atmospheric pressure, default relaxation protocol and the protein–ligand complex was simulated for a period of 50 ns. Binding energies of the molecules to receptor were calculated using the Prime MMGBSA module.^35^

### 2.21. Statistical analysis of biological assays

All results were presented as means ± standard deviation (SD) from triplicate experiments performed in a parallel manner. Data was compared using one way ANOVA (Tukey's and Dunnett's multiple comparisons test. Chi square (*X*^2^) test was used to analyze categorical data. Kruskal–Wallis test followed by Dunne's multiple comparison test were used for comparing miRNA data. Correlations between parameters were determined by Spearman correlation. All statistical calculations were performed using the computer program GraphPad Prism-8.2 (GraphPad Software, CA, USA). All comparisons were made relative to untreated controls. A statistically significant difference was considered at *P* < 0.05.

## 3 Results

### 3.1. Cell viability assay for HUVECs and EA.hy926

**Fig. 1:**
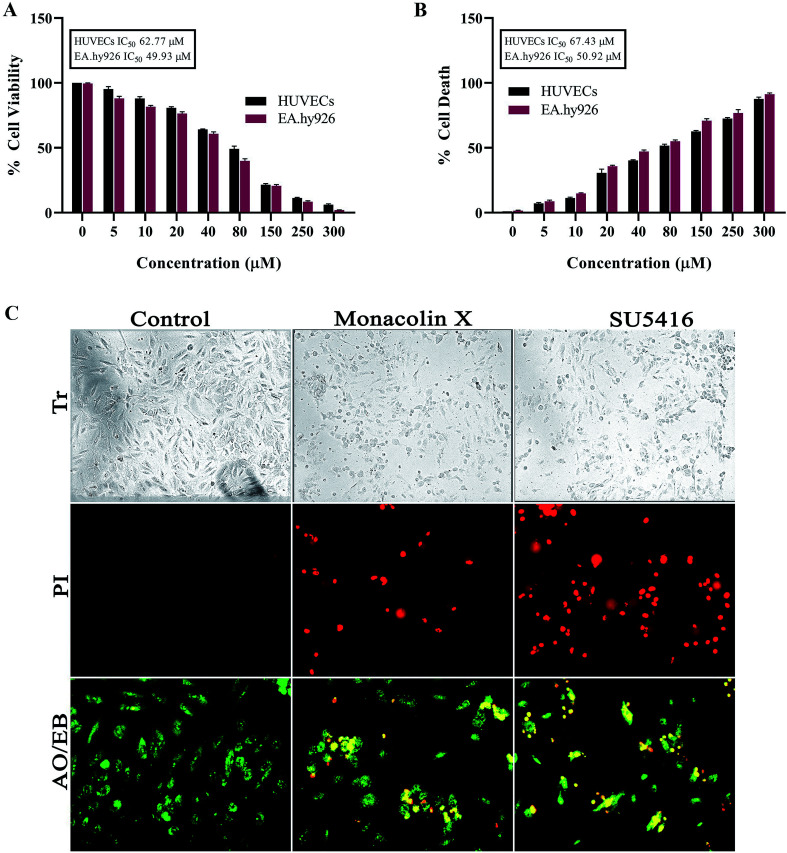
(A) Represents WST assay to check for antiproliferative and cytotoxic nature of monacolin X on human endothelial cells-HUVECs and EA.hy926. All the cells were treated with monacolin X at various concentrations (0–300 μM) for 24 h. (B) Cell membrane integrity by release of lactate dehydrogenase (LDH) activity by LDH assay. HUVECs and EA.hy926 cells were treated with monacolin X at various concentrations (0–300 μM) for 24 h. LDH released into the medium was measured along with blank, untreated cells (0 μM), had low LDH release in media and whereas treated cell had dose dependent release of LDH. (C) The morphological analysis of HUVECs treated with monacolin X at IC_50_ concentration (62.77 μM) for 24 h. Control, monacolin X treated and SU5416 treatment respectively. Morphological changes of control and monacolin X treated HUVECs cells evaluated with PI staining by fluorescence microscopy. The percentage of necrotic nuclei after 24 h treatment with monacolin X treated increased enormously, as revealed by nuclear condensation and fragmentation. Apoptotic and nuclear morphological changes in HUVECs cells treated with monacolin X evaluated with AO/EB dual staining.

As endothelial cell proliferation is important and necessary for angiogenesis, we investigated the inhibitory effect of monacolin X by WST method, where the monacolin X was treated with increasing concentrations (0–300 μM for 24 h). Results have shown dose dependent cytotoxicity on cell culture and treatment resulted in IC_50_ values for the HUVECs and EA.hy926 cell culture at 62.77 μM and 49.93 μM respectively (Fig. 1A), SU5416 was used as the positive control. The cell viability can also be measured by lactate dehydrogenase (LDH) release. The dead cells which lose their membrane integrity, will release the enzyme present inside the cell leaks out and its activity is measured externally indicating the leakage of LDH in the culture media after treating at different concentrations of monacolin X. Exposures to monacolin X elevated LDH leakage after 24 h of treatment. The treatment on HUVECs and EA.hy926 cells resulted in cytotoxicity (Fig. 1B). Treatment with monacolin X has shown shrunken cell morphology and irregular cell morphology along with a significant reduction in cell number in a dose dependent manner (Fig. 1C). This was observed using bright field phase contrast microscope.

### 3.2. Morphological evidence of apoptosis using PI & AO/EB dual staining on monacolin X treatment

Propidium iodide (PI) stain is used to show the morphological changes under a fluorescence microscope. PI staining exhibited brightly labeled PI+ pycnotic nuclei in the dead cells while viable cells remained unstained. The percentage of apoptotic nuclei after treatment with IC_50_ concentration of monacolin X considerably increased when compared to control (untreated cells) in HUVECs cells alike standard drug (SU5416) control effect. This lead to altered morphology such as nuclear fragmentation and chromatin condensation. Cells were scored at random felids and classified into apoptotic and non-apoptotic cells based on their nuclear morphology (Fig. 1C).

Further, HUVECs cells were treated with IC_50_ concentration of monacolin X and SU5416 (10 μM), showed significantly increased levels of apoptotic cells, in contrast to the untreated cells where no apoptotic cells were noticed. This was evidenced by Acridine Orange/Ethidium Bromide (AO/EB) differential staining method (Fig. 1C). The stained cells were categorized as viable (light green), early apoptotic (yellow fluorescence and condensed chromatin), late apoptotic (orange fluorescence) and non-viable cells (red colored fluorescence).^36^ The early apoptotic cells with nuclear margination and chromatin condensation were indicated in yellow colour and late apoptotic cells with fragmented chromatin were indicated in orange colour in monacolin X and SU5416 treated cells. However, the control cells have shown intact nuclear architecture, the monacolin X treated cells have shown membrane blebbing, condensed nuclei, and apoptotic bodies.

### 3.3. Effect of monacolin X on HUVECs tube formation ability

**Fig. 2:**
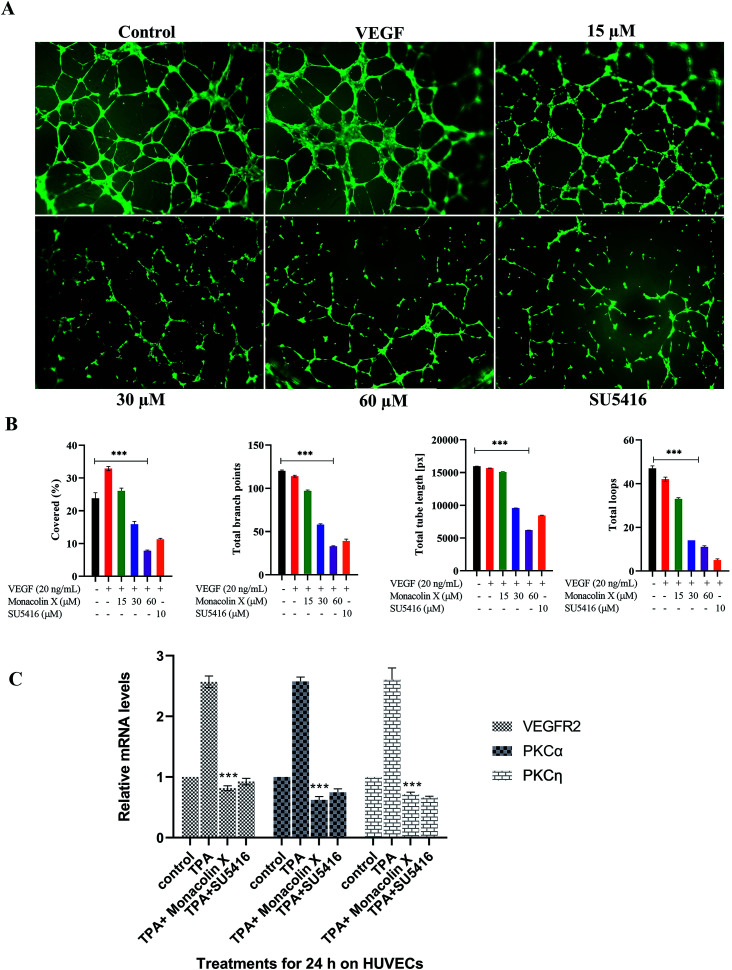
(A) Measurement of angiogenesis with the tube formation assay. HUVEC tube formation was tested with monacolin X at 3 different concentration (15 μM, 30 μM and 60 μM) and compared with standard SU5416 (magnification at 4×). (B) Total tube length (measured in pixels, px), number of branching points, number of total loops and percentage covered area (%) were measured. (C) RT-PCR gene expression results for HUVECs treated with monacolin X in presence of 10 nmol L^−1^ TPA (positive control). HUVEC, human umbilical vein endothelial cells. Each value represents three technical replicates of each of three biological replicates. Statistical significance of the relative normalized expression of monacolin X as compared to the non-treated cells is represented on the graph. β-actin gene was used as the reference gene for normalization and to calculate the relative expression based on 2-ΔΔCt. Data shows significantly different from positive control to that of monacolin X at ***P < 0.001.

Tube formation of endothelial cells is another key factor for angiogenesis, as endothelial cells can spontaneously form a 3D tubular capillary-like network on Matrigel cultures. As shown in (Fig. 2A), monacolin X was treated at 3 different concentration (15 μM, 30 μM and 60 μM) on HUVCECs cells and was able to inhibit HUVEC tube formation at even the lowest concentration, a similar pattern was observed in case of SU5416 treatment. VEGF was taken as positive control where it formed tubes completely, vessel size and thickness was also better compared to just the control and the tube inhibition were seen in the dose dependent manner in case of monacolin X, completely suppressed tube formation at its IC_50_. The bar chart showed the quantitative data, by counting the loop numbers, total tube length, branching points and area covered all these parameters significantly reduced when compared to the control group and similar pattern was observed in of standard drug SU5416 (Fig. 2B).

### 3.4. RT PCR for HUVECS

Later, the inhibitory effect of monacolin X on the VEGFR2 expression and the role played by PKC in the regulation of angiogenesis signaling were studied. This was achieved through an experiment which stimulated the PKC activity through an activator, PKCs phorbol ester (TPA). The obtained mRNA expression levels for TPA-treated cells had elevated expression of the activator PKCa when compared with control. A significant range of inhibition of the PKCa expression was seen in the IC_50_ concentration of monacolin X and SU5416 (4 μM) treated HUVEC cells which were pre-treated with TPA. This in turn resulted in partial suppression of PKCη levels which is shown in Fig. 2C. The TPA treatment resulted in increased levels of VEGFR2 mRNA, while the monacolin X treated have shown reduced levels of it. The inhibition of VEGFR2 was critically influenced by treatment with monacolin X. These results strongly suggest that monacolin X's antiangiogenic activity by modulating PKC activity, especially PKCa-mediated activation of VEGFR2.

### 3.5. Vascular sprouting assay

**Fig. 3:**
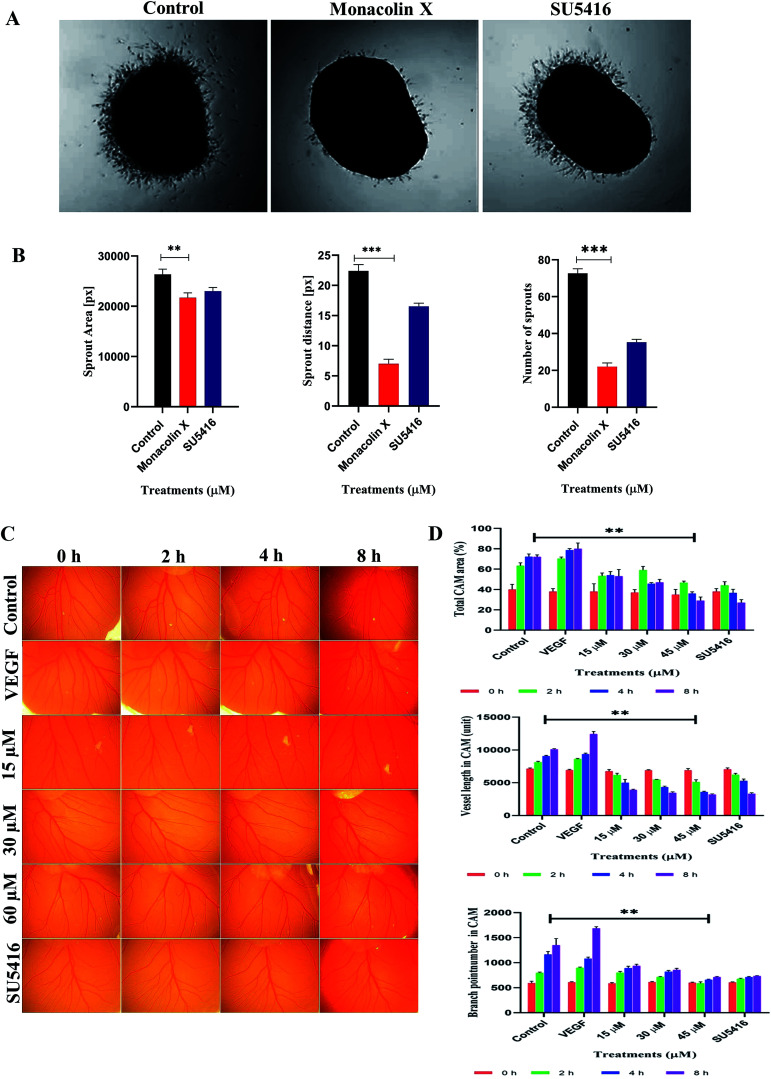
(A) Monacolin X inhibited endothelial cell sprouting in an aortic ring assay. Aortas were harvested from 12 day old chick and cut into 1 mm slices, which were then placed in 12-well plates containing matrigel. The rings were photographed and analyzed. The endothelial cell sprouting was abundant in the control aortic rings (A) but not in the rings treated with monacolin X and SU5416 suppressed the endothelial cell tube formation. All the experiments were done with the presence of VEGF (20 ng mL^−1^), (B) shows graphs for number of sprouts and length of capillary sprouts (μm). (C) Representative vascularization of the chorioallantoic membrane (CAM Assay) following 8 h of incubation with monacolin X at (15 μM, 30 μM and 60 μM) and compared to control and positive control (VEGF 20 ng mL^−1^). (D) shows the mean vessel area as a percentage of the total area, mean vessel length and mean number of branch points were obtained by Wimasis – Wimcam software. Three independent experiments were performed and the results were taken under (10×) magnification and. Each value was presented as means + SD (*n* = 3). *p < 0.05, **p < 0.01, ***p < 0.001 compared with control (one-way ANOVA).

Chick aortic ring assay was performed further to check the ability of monacolin X in suppressing the vascular sprouting. In control, the aortic rings in Matrigel prompted the growth of vascular sprouts out of the aortic wall, establishing a dense network of tubular vessel-like structures while in treatment with IC_50_ concentration of monacolin X there was an efficient inhibition (Fig. 3A). Accordingly, monacolin X treated aortic rings exhibited a significantly reduced number of vascular sprout and spout capillary length at day 4 of incubation in comparison to vehicle-treated controls. The SU5416 treated group further showed a small amount of vascular sprouting compared to monacolin X treated (Fig. 3B).

### 3.6. CAM assay

The CAM assay, a standard *in vivo* angiogenesis assay, shows vascular sprouting or angiogenesis (Fig. 3C). Chick embryos incubated with monacolin X at different concentration 15 μM, 30 μM and 60 μM for 8 h Matured blood vessels were seen in untreated embryos, while VEGF (20 ng mL^−1^) was used as positive control exhibiting the prominent blood vessels formation. The quantitative estimation of mean vessel area as a percentage of the total area, mean vessel length and mean number of branch points for egg yolks treated with monacolin X at different intervals of time (0 h, 2 h, 4 h, 8 h) the quantitative data clearly showed significantly decreased in total area, mean vessel length and mean number of branch points parameters compared to the control group and similar pattern was seen in the SU5416 treatment group (Fig. 3D). These results further suggest that monacolin X is very good antiangiogenic drug candidate.

### 3.7. Assessment of ISV

**Fig. 4:**
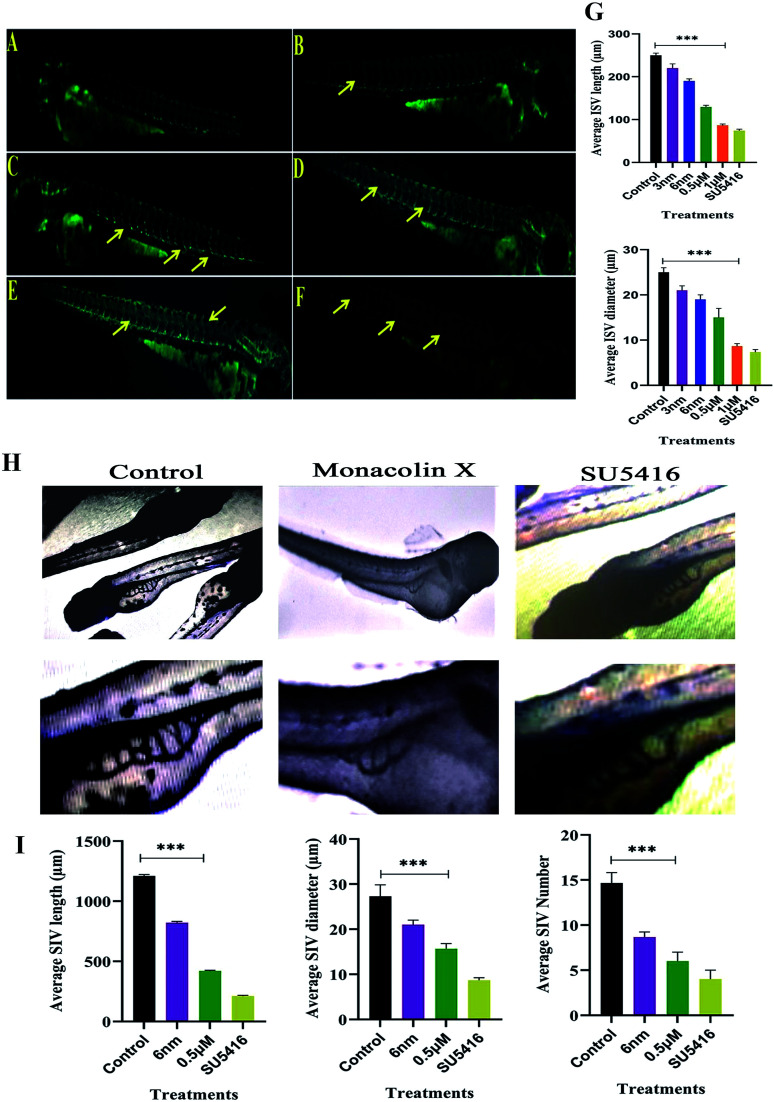
Represents panel of transgenic Tg(Kdr:EGFP)/ko1 zebrafish embryos that show green fluorescent protein (GFP) expression in endothelial cells. (A) Fluorescent images of ISV in the zebrafish embryos incubated with 0.1% DMSO (Ctrl: vehicle-control), (B–E) monacolin X 3 nm, 6 nm, 0.5 μM and 1 μM for 48 h. (F) SU5416 was used as the positive control. Yellow arrow: deformed ISV. (G) Average ISV length in (μm) and average diameter (μm). (H) Shows SIV basket stretching into the posterior yolk extension in case of control and while in monacolin X and SU5416 treatment showed significant reduction in SIV length, diameter and number of SIV. Quantification of ISV and SIV area was determined by using the NIH Image J program. (I) Shows average SIV length in (μm) and average diameter (μm) and number of SIV. All values are presented as means ± SD (*n* = 6). **p < 0.01, ***p < 0.001 compared with control (one-way ANOVA).

The assumption explained above was tested in zebrafish model system. In recent times, zebrafish which is a vertebrate system is being used for drug screening and also a classic model for angiogenesis. ISVs develop from the aorta (Fig. 4A), starting at the 24-somite stage (21 hpf)^37^ and runs between each pair of somites, and connects to the dorsal longitudinal anastomotic vessel.^38^ VEGF is articulated intensely between 18 and 19 hpf in zebrafish embryos. In order to determine and establish monacolin X effect before VEGF expression, it should be treated at 8–10 hpf (gastrulation stage) to visualize its inhibitory effect and continued till 48 hpf. This data suggests that monacolin X partially inhibited angiogenesis when administered with a concentration range of 3 nm, 6 nm, 0.5 μM and 1 μM (Fig. 4B–E) and SU5416 (Fig. 4F). Average ISV length in control was 245 μm and the treatment groups range from 220 μm to 90 μm and in case of SU5416 treated showed 70 μm. Slight impairment of ISV formation was observed in monacolin X treated group even at low concentrations. However, at higher concentration, severe defects in ISV formation in maximum embryos was seen with short growth of blood vessel in the ventral tail region and by the lacking of the formation of the angiogenic vessel.

ISV diameter in the control was 24 μm and in the treatment, groups range from 22 μm to 9 μm and in case of SU5416 treated showed 7 μm thus suggesting that monacolin X significantly reduced both ISV length and diameter in the dose dependent manner (Fig. 4G).

### 3.8. ALP staining (SIV)

After dechorionation at 24 hpf, the embryos were treated for 48 h with 0.5 μm of monacolin X along with 4 μM SU5416 (Fig. 4H). The inhibition of the vessel formation was assessed at 72 hpf. Both standard drug and monacolin X treated showed well established results of vessel inhibition thereby terminating angiogenesis at their particular concentrations. However, at lower concentrations, like 3 nm and 6 nm, the inhibition lasted only for 24 h of the treatment as the vessels started to recover and carryout angiogenesis successfully. While at higher concentrations of 1 μM to 10 μM, the angiogenesis was totally terminated resulting in the death of the embryos treated. This assumption was also tested in tg(Kdr:EGFP)/ko1 zebrafish as a prominent model system. The average SIV length in control was 1200 μm and in monacolin X treated was 450 μm respectively while in SU5416 treated it was found to be 200 μm the similar dose dependent reduction pattern was observed in the number of SIV and diameter of ISV formed (Fig. 4I). Thus from this assay, we could say the monacolin X has significantly reduced both ISV and SIV proving to be a potent antiangiogenic drug.

### 3.9. Trans migration and invasion assays

**Fig. 5:**
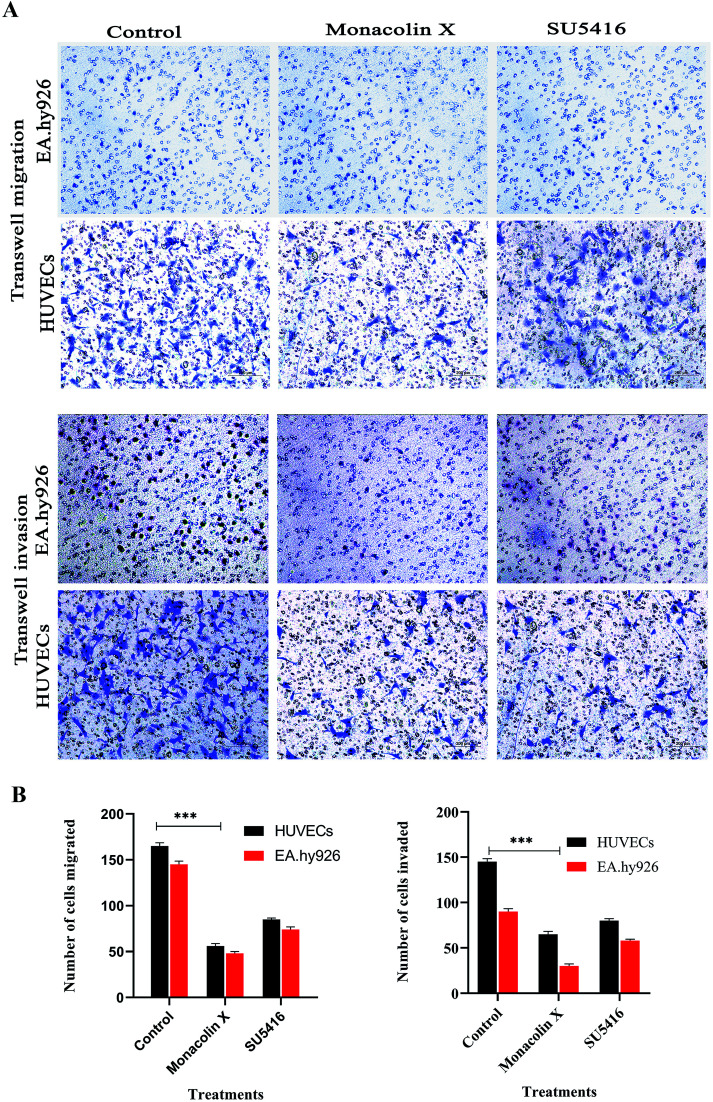
(A) Transwell migration and transwell matrigel invasion using HUVECs and EA.hy926 showed significant decrease in migration and invasion rate post monacolin X and SU5416 treatment compared to that of control. (B) Migrated and invaded cells were counted in five random fields and three independent experiments were performed and graph shows the number of migrated and invaded cells, the results were taken under (10×) magnification and the results were represented as mean ± SD (*n* = 5); ***p < 0.001 compared with control (one-way ANOVA).

The process of migration and invasion being the two most important steps in blood vessel formation during angiogenesis, clear assays of migration and invasion were conducted to detect these occurrences through modified Boyden chamber. Post the 12 h incubation, the upper surface of the transwell chambers was thoroughly wiped with cotton swabs and invading cells were fixed post which 0.05% (w/v) crystal violet solution was used for staining the cells. The cells count post the procedure was expressed in percentage of cell invasion with a referential control value of PBS. The results demonstrate that monacolin X only inhibited HUVECs migration and invasion when treated at IC_50_ concentration of monacolin X, which was incomparable to positive drug control SU5416 (Fig. 5). Thus suggesting the monacolin X has the ability to inhibit the migration and invasive property's which is a very crucial activity in case of tumor angiogenesis.

### 3.10. Wound healing assay

**Fig. 6:**
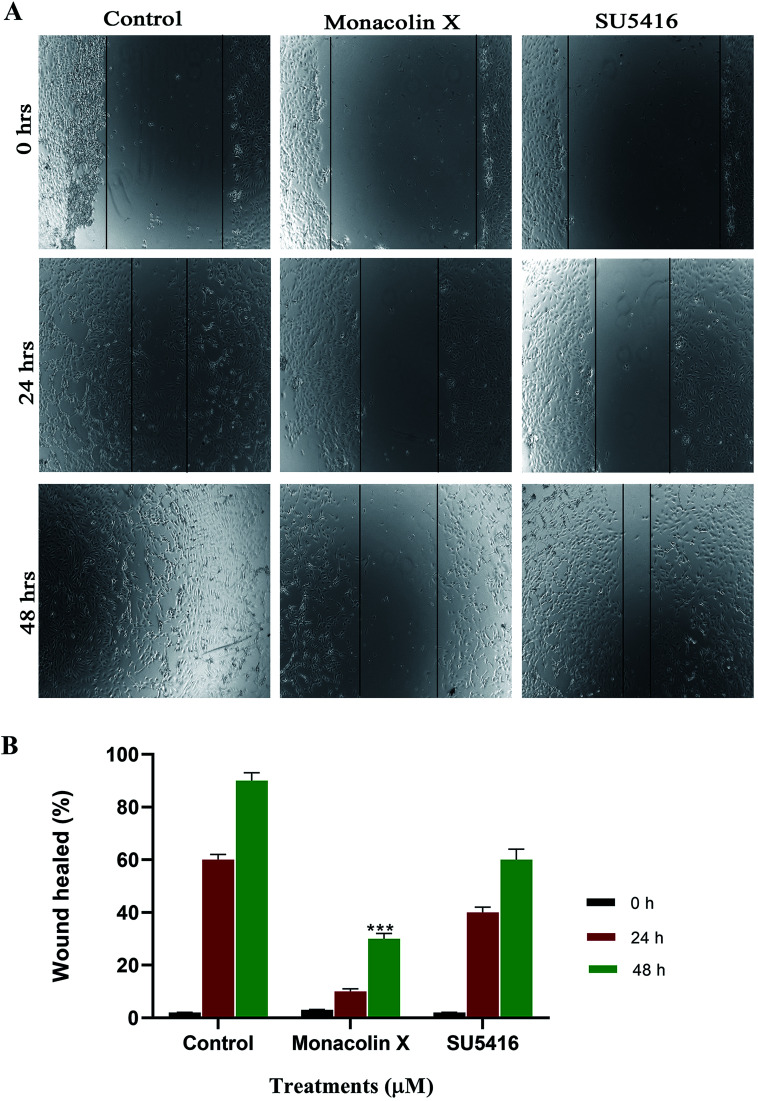
(A) Shows the antimigratory property of monacolin X and SU5416 treated on HUVECs human endothelial cells for 48 h. Significant differences between the control groups and treated groups in terms of the number of migrated cells were noted at 24 h and 48 h. Monacolin X treated group showed less number of migrated cells compared to control endothelial cells. Magnification (4×). (B) Experiments were performed in triplicate and the data are expressed as mean ± SD (*n* = 3); ***p < 0.001, as compared to control group were considered as significant.

The endothelial cell migration is one of the key steps of the angiogenesis process. In order to check the migration of endothelial cells (HUVECs) and to ascertain the inhibitory effect of monacolin X and SU5416 on endothelial cell migration. There was significantly reduced migration on treatment with monacolin X and SU5416 (4 μM), when compared to untreated cells (Fig. 6A), indicating the IC_50_ concentration of monacolin X having a very good anti-migratory property. The assay was carried out for 48 h and microscopic observation was taken at 0 h, 24 h and 48 h. However, both the monacolin X and SU5416, did not show significant wound closure in HUVECs compared to untreated control cells. The extent of wound healing was quantified and presented as a histogram (Fig. 6B). The results showed an almost threefold increase in the % wound healing in case of HUVECs untreated compared to that of treated.

### 3.11. Immunofluorescence (IF) analysis

**Fig. 7:**
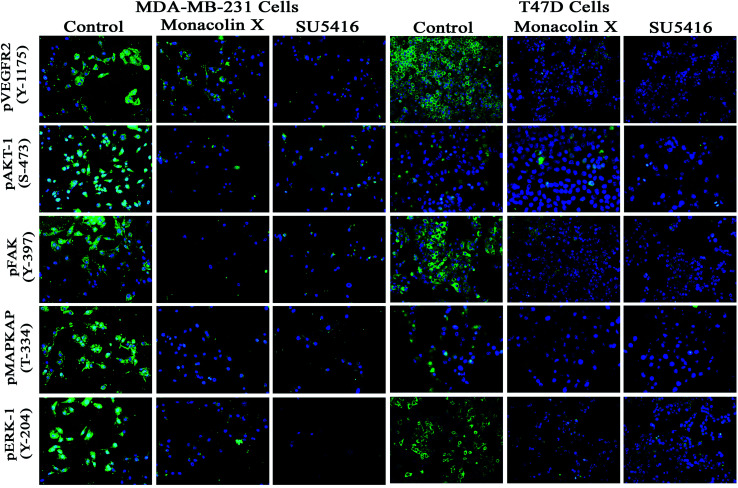
Represents immunofluorescence staining for expression of anti-pVEGFR- 2, pAKT1, pFAK, pMAPKAPK2 and pERK1 antibody marker (green), and nuclear (blue) counterstain. Control group showed significant expression but on treatment with monacolin X and SU5416 treated for 24 h showed significant decreased expression of all the phosphorylated forms in both MDA-MB-231 and T47D breast cancer cell line magnification (10×). All the experiments were done with the presence of VEGF (20 ng mL^−1^) experiments were performed in triplicate as compared to control group were considered as significant.

To understand the inhibition of monacolin X at IC_50_ concentration on tumor angiogenesis in breast cancer through VEGFR2 downstream signaling pathway, immunofluorescence (IF) analysis was performed for the downstream cascade of the VEGFR2 signaling pathway. The proteins expression levels on monacolin X treated was high in MDA-MB-231 (high metastatic) and T47D (low metastatic) breast cancer cell line when compared to the control group. IF analysis revealed that in untreated cells pVEGFR2, pAKT1, pFAK, pMAPKAPK2 and pERK1 were significantly high compared to that of monacolin X treated group. In control, the expression were showing high intensity of green fluorescence for pVEGFR2 (expressed in cell membrane), pAKT1 (cell membrane and nucleus), pFAK (cell membrane, cytoskeleton and nucleus), pMAPKAPK2 (cell membrane and nucleus) and pERK1 (diffused in nucleus) (Fig. 7). The treatment with monacolin X showed significantly decreased protein expression with very low green fluorescence, a similar pattern was observed in SU5416 treatment.^39^

### 3.12. Effect of drug on tumor weight, tumor volume and tumor incidence in control and experimental group of animals

**Fig. 8:**
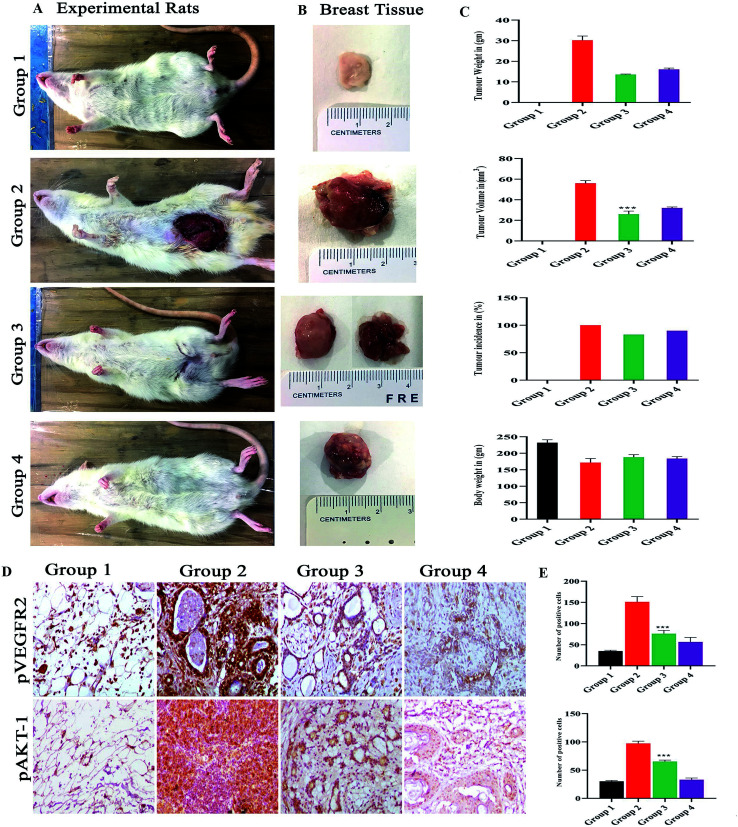
(A) Shows the representation of rats for 4 different groups of experimental rats and (B) the mammary gland morphology for all the groups. Where the control group 1 control shows normal breast morphology and group 2 DMBA induced group shows an incased tumor formed group 3 monacolin X treated post tumor induction and group 4 SU5416 treated group. Whereas the graphs (C) post treatment with monacolin X and SU5416 shows reduction in the tumor weight (gm) and volume (mm^3^), body weight (gm) and tumor incidence (%). (D) Shows the immunohistochemical analysis for tumor angiogenesis and survival status *via* pVEGFR2 and pAKT1 expression were significantly increased in the group 2 (DMBA) when compared to group 1, the treated group 3 and 4 (DMBA + monacolin X) (DMBA + SU5416) showed decreased expression levels of these proteins. The results were taken under (10×) magnification and the results were represented as mean ± SD (*n* = 3); ***p < 0.001 when compared with DMBA group. (E) Shows the histogram for the number of positive cells for pVEGFR2 and pAKT1.

The group I was treated with corn oil (vehicle control) there were no pathological changes observed in the mammary gland region and this group served as control, the tumor representative images are shown in Fig. 8A and B. In group II, the tumor weight was 32.33 ± 12.5 g, tumor volume was 59 ± 12.12 mm^3^ and incidence of tumor was six out of six animals, while when treated with monacolin X in group III, there was a significant decrease to 13.63 ± 1.4 g and 26.6 ± 7.37 mm^3^ in both the parameters, similar trend seen in group IV, was administered with SU5416 showed decreased tumor weight and volume. At the end of the study, the number of tumors was calculated in group II, group III and group IV accordingly. Group II has reported 16 tumors, group III were reported with 6 tumors while group IV with 8 tumors (Fig. 8C).

### 3.13. Immunohistochemistry

The immunohistochemical analysis revealed the monacolin X acting as inhibitor for VEGFR2 by downregulating the pVEGFR2 and pAKT1 expression indicating the antiangiogenic nature of monacolin X (Fig. 8D). Group II (DMBA) showed significantly increased expression of VEGFR2 in endothelial cells (membrane) and tumor cells (nuclei, cytoplasm, and membranes) while, pAKT1 expression was observed only in tumor cells (cell membrane and nucleus) when compared to group I. The treated group III (DMBA + monacolin X) and group IV (SU5416) showed decreased expression levels of these proteins, suggesting the potent antiangiogenic activity of monacolin X in DMBA induced mammary carcinogenesis. The histogram shows number of positive cells for pVEGFR2 and pAKT1 (Fig. 8E).

### 3.14. Docking analysis and molecular dynamic simulation of VEGFR2 with monacolin X and standard drugs SU5416

**Fig. 9:**
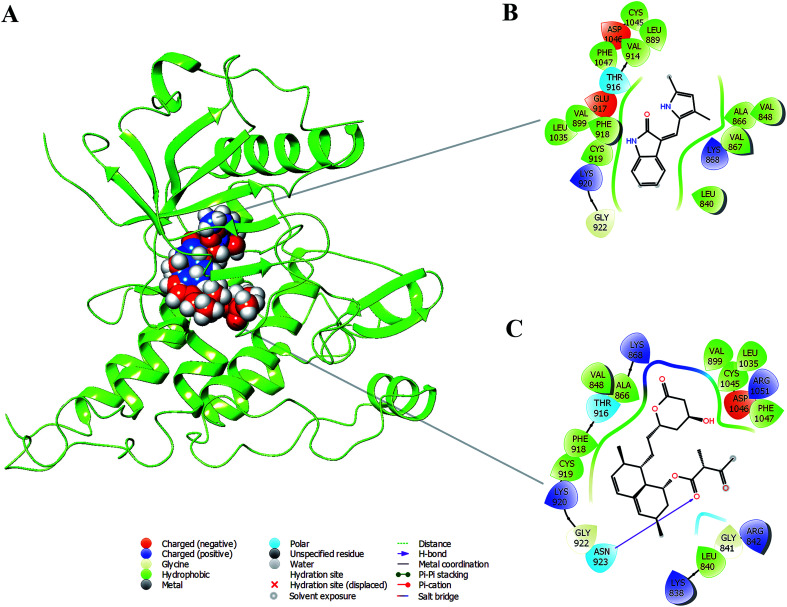
(A) Binding modes of SU5416 and monacolin X inside the active pocket of the VEGFR2 (B) & (C) LigPlot depicting the interaction profiles of SU5416 and monacolin X with the active pocket amino acids after docking studies.

Based on the above *in vitro* and *in vivo* experimental results it was confirmed that monacolin X had anti-angiogenesis activity through inhibiting VEGFR2 pathway. To further understand about where exactly the monacolin X is binding in the VEGFR2 active pocket, computational methods like docking and simulation studies were executed. The computational approach would be an added advantage to experimental studies in analyzing the data and generating reliable results. Docking studies were carried out on monacolin X and standard drug SU5416 against the receptor using glide docking module with XP precision mode. Standard drug accommodated well inside the active pocket but failed in making interactions with the pocket amino acids. Monacolin X also fitted well inside the active pocket by establishing one hydrogen bond between a =O group of the molecule with NH group of Asn 923 residue of the receptor (Fig. 9A–C). Energy calculations were performed to the two complexes obtained from docking studies to calculate the relative binding affinity of the ligands against the receptor. The docking scores along with energy calculations of the two complexes were reported in Table 1. These two complexes were further analyzed using the Desmond simulation studies to calculate the ligand binding efficiency with the amino acids present in the active pocket. The deviations and fluctuations in the amino acids during the simulation were analyzed using the trajectory files. Deviations in the complex were represented in the RMSD graph and fluctuations in the form of RMSF graph. By the end of 50 ns simulation run time, the standard drug in complex with the protein produced two hydrogen bonds with Cys 919, which is the crucial amino acid in VEGFR2 inhibition.^40^

**Table 1:** Denotes Qikprop, docking and simulation based analysis of SU5416 and monacolin X molecules^a^

Molecule	QikProp analysis					G-score	BMDS		AMDS	
	CNS	MW	QP log BB	%HOA	Lipinski rule		DG-bind (kcal mol^−1^)	H-bond	DG-bind (kcal mol^−1^)	H-bond
SU5416	0	238.28	−0.32	100	0	−6.4	−39.93	No	−45.32	Cys 919
Monacolin X	−2	418.52	−1.557	86.51	0	−6.7	−47.42	Asn 923	−48.62	Cys 919, Asn 923

a CNS: predicted central nervous system activity on a −2 (inactive) to +2 (active) scale. MW: molecular weight of the molecule. QP log BB: predicted brain/blood partition coefficient −3.0 to 1.2. %HOA: predicted human oral absorption on 0 to 100% scale. DG-bind (kcal mol^−1^): prime energy. H-bond: hydrogen bond interaction.

**Fig. 10:**
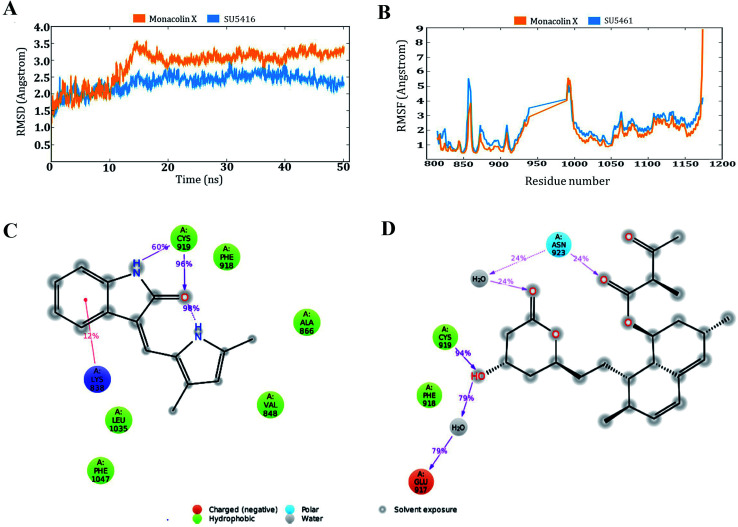
Molecular dynamic simulation results of the SU5416–receptor and monacolin X–receptor complexes: (A) deviations made by the receptor in the presence of SU5416 and monacolin X during 50 ns of simulation time. (B) Fluctuations made by the receptor amino acids in the course of the simulations. (C) & (D) Binding profiles of the SU5416 and monacolin X by end of simulations.

Monacolin X showed one hydrogen bond interaction with Cys 919 and Asn 923 amino acids of the receptor, by the end of the simulation run. The deviations made by the receptor in the presence of standard drug were in 1.5–3.0 Å range and the majority of the deviations were observed in the 2.0–2.5 Å range. Whereas, in receptor–monacolin X complex, the protein deviations are higher compared to the receptor–standard drug complex, observed in 1.5–3.5 Å range. Deviations in the complex were reported starting from 1.5 Å in the initial time frames and attained a maximum deviation of 3.5 Å by the end 18 ns, further decreased to 3.0 Å and continued around 3.0–3.5 Å range till to the end of simulations. Receptor amino acids fluctuations are marginally more in SU5416 complex compared to monacolin X complex. In both monacolin X and SU5416 complexes, fluctuations range above 3.0 Å was majorly observed at three regions, namely 850–860, 940–1000 and at the tail end. Tail end region can be omitted because of their flanking behavior and because of that, they produce high fluctuations. Docking study plots and simulation study graphs of the two complexes were shown in (Fig. 10A–D).

## 4 Discussion

For angiogenesis to take place, endothelial cells must be activated and start to proliferate, migrate and invade through breaking the basement membrane and extracellular matrix to differentiate and yield a new vessel capillary. To inhibit angiogenesis any of these steps can be considered as a pharmaceutical target.^41^ Angiogenesis inhibitors are likely to change the face of medicine, accelerating as an attractive approach for treating cancer and many other angiogenic-dependent diseases.^42^ The vast majority of the natural compounds which are been used as angiogenesis inhibitors, which are isolated majorly from terrestrial microorganisms and plants, mainly due to their high availability and known as indigenous medicine.^43^ Marine-derived antiangiogenic molecules have been less explored, where recent scientific attention is being held for the development of new marine-derived drugs. Many marine organisms produce metabolites that allow them to adapt and survive in extreme environments, marking them as unique molecules of the highest interest for drug discovery.^44,45^

Monacolin X, a secondary metabolite of marine sponge associated fungus reported previously^13^ demonstrated that monacolin X could significantly inhibit proliferation, migration, invasion and tube formation abilities, and apoptosis inducing ability and downregulating the PKCα *via* VEGFR2 in HUVECs *in vitro*. Monacolin X suppressed endothelial cell proliferation in a concentration-dependent manner and elicited its maximal effect at 300 μM. In this study, the cytotoxic activity of monacolin X against HUVECs and EA.hy926 showed a decrease in the number of viable cells due to an increase of cell death and/or decreased cell proliferation, in a dose-dependent manner and induction of cell death through morphological alterations such as cell shrinkage, membrane blebbing, rounded and detached cells.^46^ Apoptosis is programmed cell death, an important physiological process for the maintenance of tissue homeostasis and plays a pivotal role in the pathogenesis of various diseases.^47^ Apoptosis occurs without eliciting a local inflammatory response, in the case of cancer therapy, one of the most important aspects is to induce apoptosis and kill cancer cells and the endothelial cells. Therefore, apoptosis was confirmed by AO/EB staining, where the IC_50_ (62.77 μM) value was used to evaluate the apoptosis induced by monacolin X. The characteristics of late apoptosis include loss of membrane integrity and uptake of PI. Monacolin X treatment indicated that more than 40% of cells were dead when compared to the standard control of SU5416.

Endothelial cell migration is a key step during the process of angiogenesis. Monacolin X treatment reduced the number of migrated cells, a characteristic feature of different angiogenic phenotypes of ECs. This could be due to the effect of monacolin X which arrested the phosphorylation of FAK.^48^ Furthermore, monacolin X treatment blocked HUVEC invasion induced by VEGF. Similar results were reported by Rodríguez-Nieto *et al.*, 2001 on aeroplysinin-1, a brominated compound isolated from a marine sponge which showed reduced invasion.^49^

Monacolin X treatment on HUVEC's tube formation induced by VEGF significantly reduced due to antiangiogenic activity of monacolin X depending on the prevention of capillary-like tube formation rather than proliferation. This inhibitory effect of monacolin X on the morphogenesis of endothelial tubes is not due to cytotoxicity and is exerted at even low concentrations. In addition, among angiogenesis assays, CAM assay is a well-established and widely used model to confirm ex vivo anti-angiogenesis. It was observed that monacolin X inhibited developing vessels of the CAM, probably because of the induction of apoptosis in the vascular cells and their progenitors. This might be due to the fast disorganization and the loss of integrity of the vascular wall in the preexisting vessels, as revealed by direct observation.^50^

Aortic ring endothelial cell sprouting assay was used to confirm the inhibition of angiogenesis on monacolin X treatments resulted in a dose and time dependent decrease in capillary sprout formation. The sprouts growth were shorter around the ring and only a few cells were migrated into the matrix in monacolin X treated group compared to normal, indicating the effect of monacolin X on blocking neo-vascularization *in vivo*. In order to check whether the angiogenesis suppression on chick aortic ring was due to the cytotoxic or anti-proliferation effects of the monacolin X and was withdrawn after its exposure to aortic rings.^51^ Further, zebrafish embryos treated either with monacolin X or SU5416 for 72 h showed a remarkable anti-angiogenic phenotype, demonstrating reduced ISV and SIV formation. The exposure to monacolin X during the gastrulation period had a significant effect on the formation of a new blood vessel in ISVs compared to post gastrulation treatment. This indicates that the monacolin X did not cause any normal vasculature disruption, but likely inhibited the formation of new blood vessels by blocking the angiogenesis signaling pathway during angiogenesis.^15^ These results clearly suggest that incubation with monacolin X suppresses several features of angiogenesis in HUVECs *in vitro* and in zebrafish *in vivo*. Similarly, many natural compounds like Sesterterpenes, Aeroplysinin-1, Elaiophylin and curcumin have shown to inhibit the angiogenesis process.^52–56^

Our results strongly suggest that monacolin X inhibits TPA-induced PKC activation. Additionally, the mRNA expression of VEGFR2 was found to be downregulated in HUVECs cells post treated with monacolin X. The inhibition of PKCa *via* VEGFR2 in the presence of monacolin X in HUVECs cells shows PKC-mediated inhibition of angiogenesis. The results propose that monacolin X regulates the signal transduction pathway involved in the activation of TPA-responsive PKC isozymes, especially PKCa, in down regulating VEGFR2 expression in HUVECs.^15^

The VEGFR2 in tumor angiogenesis plays a major role and it's been upregulated in various cancer lung, colon, uterus, ovarian cancer, as well as breast cancers.^57^ VEGF/VEGFR2 was found to be essential for MDA-MB-231 and T47D to cell survival and migration,^58,59^ this was similar for leukemia and prostate cancer cells.^60,61^ The vascular endothelial growth factor (VEGF) with VEGFR2 causes cell proliferation, survival, migration, capillary-tube formation and permeability increase that occurs in rapidly growing tumors.^62,63^ The VEGF subtypes VEGFA, VEGFC, VEGFD, and VEGFE can activate VEGFR2 (Flk-1/KDR). VEGFR2 is the main signal transducer responsible for angiogenesis because it has strong intrinsic kinase activity and plays a decisive role in the regulation of tumor angiogenesis.^64^ Phosphorylation of VEGFR2 is most important to regulates downstream signaling^65,66^ in turn activating Akt which helps in cell survival, p38 mitogen-activated protein kinase (p38 MAPKAP) help in actin reorganization and vascular permeability, FAK helps in cell migration and ERK1/2 in cell proliferation. To determine whether blocking the VEGF–VEGFR2 interaction will prevent the VEGF-mediated cellular proliferation, survival and migration. The monacolin X was treated along with VEGF to see the prevention of VEGF-induced proliferation of both MDA-MB231 and T47D cells and to check VEGFR2 downstream proteins in the phosphorylated form (pVEGFR2, pAkt1, pMAPKAPK2 and pERK1/2) and were quantified by immunofluorescence.^67^ This result strongly suggests that VEGF protects breast cancer cell viability *via* VEGFR2 (flk1/kdr) and monacolin X treatment is inhibiting the cell proliferation acting as VEGFR2. Similar kind of the results were reported by Zhou XA *et al.* and Tang N *et al.*, Eriocalyxin B and Gamabufotalin natural compounds inhibited VEGF-induced angiogenesis and diminished angiogenesis-dependent breast tumor growth by suppressing VEGFR2.^68,69^

Further, DMBA induced breast cancer in Sprague-Dawley rats, treated with monacolin X showed reduced tumor volume and size compared with those DMBA alone treated rats. Remarkably, the monacolin X treatment also prolonged the survival of tumor-bearing rats. Our results are in line with other studies where the antitumor effect of *in vivo* previously reported.^55,70^ In addition, the pVEGFR2 and pAkt levels were reduced in the monacolin X and SU5416 treated group in compared to that of DMBA breast cancer induced group suggesting the monacolin X is able to decrease the tumor angiogenesis process and kill both cancer cells and endothelial cells thus monacolin X could be a potent antiangiogenic VEGFR2 inhibitor. Our results were similar to Ma J *et al.* and Guan YY *et al.* showed the anthraquinone derivative emodin and Raddeanin A downregulating VEGFR2 activity in breast cancer and colorectal tumor rat model *in vivo*.^71,72^

Molecular docking based virtual screenings using a protein target with experimentally determined structures are in lead discovery for various drug developments.^73^ Molecular dynamic simulation is a standard tool for understanding physical interaction between a receptor and ligand (small molecule) in drug discovery.^74^ Previously, several studies reported the importance of Cys 919, Asp 1046 and Glu 885 as important amino acids present in the VEGFR2 binding pocket.^40^ To act as an effective inhibitor, the ligand must show predominant interaction especially with Cys 919 a crucial amino acid among the three reported. The present *in silico* study mainly focused on the interactions made by the monacolin X against the crucial amino acids during the docking and molecular dynamic simulations. SU5416, a well-known reported active inhibitor was chosen for comparative study in the work design. Docking studies assisted in gaining insight knowledge about the two molecules accommodation inside the VEGFR2 active pocket and their interactions. In the docking study, the two molecules failed to generate a hydrogen bond with Cys 919 residue. However, by the end of simulations run, both the molecules attained hydrogen interaction with the important amino acid. SU5416 showed two hydrogen bonds with Cys 919 (96% and 60%) and monacolin X showed one hydrogen bond with Cys 919 (94%). Monacolin X retained the hydrogen bond interaction with Asn 923 (39%) which was reported earlier in the docking studies. The hydrogen bonds percentage reported here reflects the number of hydrogen bonds formed during the simulation run time counting the entire 50 ns runtime as 100%. The formation or disappearance of hydrogen bonds during the simulation run was mainly because of the receptor and ligand flexibility nature. The flexibility nature between the receptor and ligand supported in generating the conformers and during the conformational changes the two molecules made interaction with Cys 919 making the complex more stable. The conformational changes in the complexes during the simulations were represented in the form of deviations (RMSD) and fluctuations (RMSF) graph. The torsion made by the single bonds present in the monacolin X during molecular simulation studies were depicted in Fig. S1.† Cys 919 residue of the receptor was found to produce a strong H-bond (94%) with the OH group of the molecule because of the minimal torsions in the single bonds attached to that particular benzene ring.

## 5 Conclusion

**Fig. 11:**
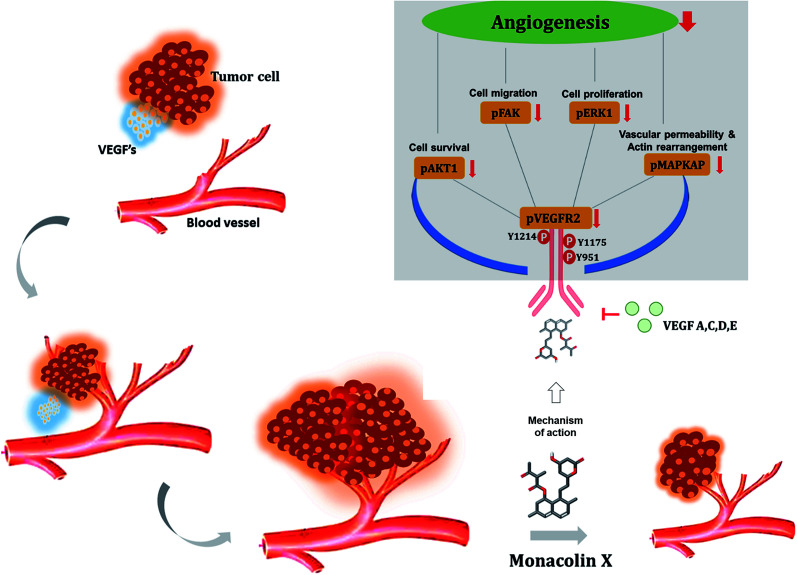
Illustrates the overall mechanism of action of monacolin X on VEGFR2 in tumor angiogenesis.

For the first time monacolin X has been studied in *in vivo*, *ex vivo*, *in vitro* and *in silico* models for explaining its action and mechanism as a potent inhibitor of angiogenesis and tumor angiogenesis acting *via* downregulating the VEGFR2 pathway upon VEGF stimulation. In addition, *in vivo* angiogenesis showed inhibition using CAM assay, aortic ring assay and SIV and ISV formation in zebrafish and in DMBA breast cancer induced models. Simulation studies also showed it to be a potent inhibitor, thus VEGFR2/Flk1/KDR is proved to be a promising target which has a high potential for further lead optimization as a chemotherapeutic agent (Fig. 11).

## Funding

The corresponding author Dr A. Madan Kumar is grateful to the Department of Science and Technology, Govt of India for the support and grant sanctioned to carryout this work under start-up research grant (young scientist) DST-SERB (SB/YS/LS-101/2014). The Sirpu Natesh Nagabhishek gratefully acknowledges to ICMR-SRF fellowship (3/2/2/7/2019/NCD-III). The corresponding author is also grateful for the grant sanctioned by Department of Biotechnology, Aquaculture and Marine Biotechnology, Govt of India under the taskforce (BT/PR11664/AAQ/3/682/2014) research grant.

## Conflicts of interest

The corresponding author declare that no conflicts of interest exist.

## Ethical approval

The informed consent was obtained for any experimentation with human subjects. The ethical clearance was given by institutional animal ethical committee with the approval number [Approval no: SU/CLATR/IAEC/IV/025/2016].

## Informed consent

For this type of study, formal consent is not required to carry out the work.

## Author's contribution

Sirpu Natesh Nagabhishek done samples collections, carried out all the experiential work, has done all data collection, interpretation and drafted the manuscript; Madan Kumar designed the study, done results interpretation and drafted the manuscript; both the authors read and approved the final Manuscript. Sambhavi B and Anandan B helped to carry out studies on HUVECs cells, while Yash T Katakia and Suvro Chatterjee provided laboratory space and helped to perform assays using EA.hy926, CAM and arotic ring; N. Nagasundaram helped to execute *in silico* studies.

## Abbreviations

HUVECs: Human umbilical vein endothelial cells
VEGF: Vascular endothelial growth factor
VEGFR2: Vascular endothelial growth factor receptor 2
ISV: Intrasegmental vessels
SIV: Sub intestinal vessels
FAK: Focal adhesion kinase
MAPKAPK2: Mitogen-Activated Protein Kinase-Activated Protein Kinase 2
ERK1: Extracellular Signal-Regulated Kinase 1
DMBA: 7,12-Dimethylbenz(*a*)anthracene
SD: Spark dawley
Cys: Cysteine
Tg: Transgenic
h: Hours
hpf: Hours post fertilization
nM: Nanomolar
μM: Micromolar
mM: Millimolar

## Supplementary Material

RA-009-C9RA05262C-s001
