# Supplementary material for: A marine sponge associated fungal metabolite monacolin X suppresses angiogenesis by down regulating VEGFR2 signaling
Source: RSC Adv. 2019 Aug 27;9(46):26646–67. doi: 10.1039/c9ra05262c (PMC9070443; doi:10.1039/c9ra05262c)
Supplement: RA-009-C9RA05262C-s001 [file RA-009-C9RA05262C-s001.pdf]

Electronic Supplementary Material (ESI) for RSC Advances.
This journal is © The Royal Society of Chemistry 2019

A Marine sponge associated fungal metabolite Monacolin X suppresses angiogenesis by down regulating VEGFR2 signaling

Sirpu Natesh Nagabhishek, Arumugam Madan Kumar*, Sambhavi B, Aanandan Balakrishna, Yash T Katakia, Suvro Chatterjee, N.Nagasundaram.

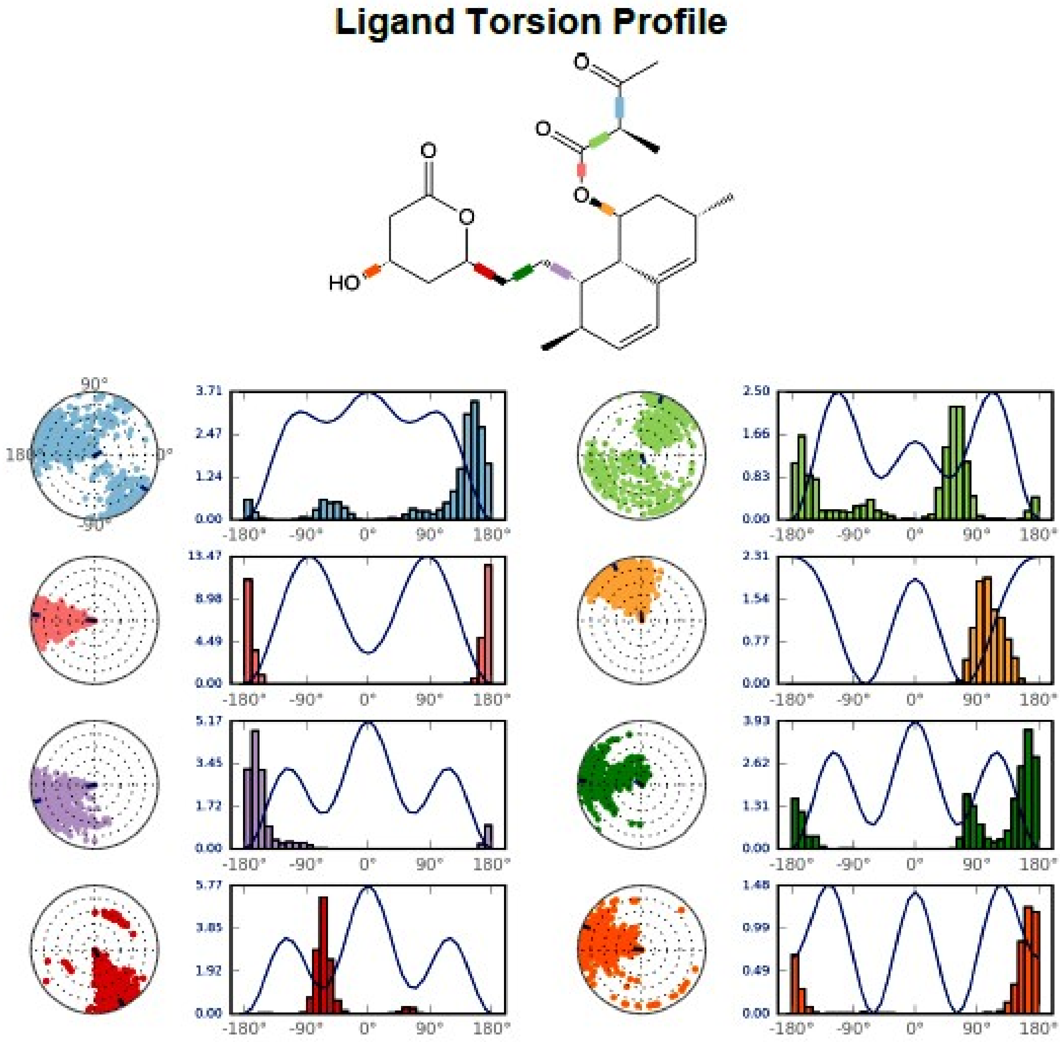

Supplementary Figure 1: Shows torsion profile for monacolin X
